# Challenges in Ultra-Trace Beryllium Analysis: Utilizing Recent Extraction Techniques in Combination with Spectrometric Detection

**DOI:** 10.3390/toxics13040289

**Published:** 2025-04-09

**Authors:** Lucia Nemček, Ingrid Hagarová

**Affiliations:** Institute of Laboratory Research on Geomaterials, Faculty of Natural Sciences, Comenius University in Bratislava, Mlynská dolina, Ilkovičova 6, 842 15 Bratislava, Slovakia; ingrid.hagarova@uniba.sk

**Keywords:** beryllium, spectrometric quantification, liquid-liquid extraction (LLE), cloud-point extraction (CPE), solid-phase extraction (SPE)

## Abstract

Beryllium (Be) is one of the most toxic non-radioactive elements on the periodic table, and its presence or intake can negatively impact both the environment and human health. Classified as a carcinogen, Be is dangerous even at trace concentrations, stressing the necessity of developing reliable methods for quantifying it at very low levels. Spectrometric techniques for quantifying Be vary in sensitivity and applicability, with inductively coupled plasma mass spectrometry (ICP-MS) being the most sensitive for ultra-trace analysis. Flame atomic absorption spectrometry (FAAS) is suitable for higher Be concentrations, but preconcentration techniques can significantly lower detection limits. Electrothermal atomic absorption spectrometry (ETAAS) provides enhanced sensitivity for low-level Be quantification, further optimized using pyrolytically coated graphite tubes and chemical modifiers such as Mg(NO_3_)_2_ or Pd(NO_3_)_2_. Effective separation and preconcentration techniques are essential for reliable Be quantification in complex matrices. Liquid-liquid extraction (LLE), including single-drop microextraction (SDME) and dispersive liquid-liquid microextraction (DLLME), have evolved to reduce the use of hazardous solvents. When combined with ETAAS, surfactant-assisted DLLME using agents like cetylpyridinium ammonium bromide (CPAB) and dioctyl sodium sulfosuccinate (AOT) achieves preconcentration factors of approximately 25, reducing LOD to 1 ng/L. Vesicle-mediated DLLME coupled with ETAAS further enhances sensitivity, allowing detection limits as low as 0.01 ng/L in seawater. Cloud-point extraction (CPE), often employing Triton X-114, facilitates Be extraction using complexing agents or nanomaterials like graphene oxide. These advancements are critical for accurately quantifying Be at ultra-trace levels in diverse environmental and biological samples, overcoming challenges posed by low analyte concentrations and matrix interferences.

## 1. Introduction

Beryllium (Be), a Group IIA element in the periodic table, is present in the Earth’s crust at an estimated concentration of 2.8–5 mg/kg [1]. This lightweight metal has a high melting point of 1287 °C and exceptional properties: it is lighter than aluminum, about 40% stronger, and approximately one-third more flexible than steel [2]. These unique physicochemical characteristics make beryllium invaluable in various alloys, where it enhances strength, increases electrical and thermal conductivity, and improves resistance to corrosion and wear. For this reason, beryllium is widely used in electrical devices, electronic instruments, telecommunications equipment, and structural components for airplanes, rockets, satellites, and nuclear reactors [3]. However, these applications contribute to the release of beryllium into the environment. Furthermore, activities such as fossil fuel combustion and ore smelting and mining generate industrial effluents that contaminate air, soil, and water bodies [4]. As a result, global beryllium contamination is rising, posing significant risks to both the environment and human health.

Beryllium is recognized as a toxic element, with reports of its toxicity dating back to the 1930s, when numerous cases of beryllium poisoning emerged in the rapidly expanding industries of that time [5]. Beryllium and its compounds can cause various skin issues, including inflammation, ulcer formation, and granulomas. One of the most serious conditions associated with beryllium exposure is berylliosis, also known as Chronic Beryllium Disease (CBD), which is characterized by chronic inflammation and granuloma formation in the lung tissues [6]. Inhalation of beryllium, particularly in the form of smoke, mist, or fine dust particles under 10 µm, can result in acute lung diseases such as bronchitis, pneumonia, and alveolar edema [7]. Moreover, animal studies have shown that beryllium and its compounds can be carcinogenic, teratogenic, and mutagenic [8]. With accumulating evidence of its carcinogenicity, beryllium-containing substances have been classified as carcinogenic to humans [5]. The International Agency for Research on Cancer (IARC) in Lyon has designated beryllium as a Group 1 carcinogen, a category reserved for agents with strong evidence of carcinogenic effects in humans [9].

To address the potential risks associated with even ultra-trace levels of beryllium, which can enter the food chain and pose significant health hazards, the ability to accurately measure such exceedingly low concentrations in environmental samples is crucial. To meet these concerns, advanced methods have been developed for the separation, preconcentration, and precise quantification of beryllium at ultra-trace levels.

While beryllium’s unique properties have made it the subject of thousands of scientific publications, only a small fraction focus on developing analytical methods for its reliable quantification. Among the available approaches, spectrometry stands out for its effectiveness in detecting this element. This article explores the widely used spectrometric techniques combined with recent extraction strategies for accurately quantifying ultra-trace concentrations of beryllium in diverse real-world samples.

## 2. Spectrometric Methods for Beryllium Quantification

Quantification of beryllium can be performed using several spectrometric techniques, each offering different levels of sensitivity and applicability depending on the matrix and analytical objectives. This section briefly reviews the most common spectrometric techniques employed for beryllium quantification.

Flame atomic absorption spectrometry (FAAS) operates on the principle that free atoms in the gas phase absorb light at specific wavelengths. In this approach, a sample solution is introduced into a flame, where it is vaporized and converted into free atoms. A light source, typically a hollow cathode lamp, emits light at a wavelength specific to the element being analyzed, such as beryllium. As this light passes through the flame, the free atoms absorb part of it at the element-specific wavelength. A detector positioned after the flame measures the amount of absorbed light, which is directly proportional to the concentration of the element in the sample. Although FAAS is less frequently used for direct ultra-trace quantification of beryllium due to its relatively low sensitivity, it is effective for samples with higher beryllium concentrations or when combined with an efficient separation and preconcentration procedure for samples with very low beryllium concentrations [10]. The instrumental parameters for FAAS-based beryllium quantification are typically configured according to the manufacturer’s guidelines. Studies often report the use of a hollow cathode lamp set to 234.9 nm and an N_2_O–acetylene flame, while a deuterium lamp is typically used for background correction [10,11,12].

Electrothermal atomic absorption spectrometry (ETAAS) works on a similar principle to that of FAAS, with key differences in the atomization process. In ETAAS, a small volume of the sample (typically a few microliters) is introduced into a graphite furnace, which heats the sample in a controlled sequence of four stages: drying, ashing, atomization, and cleaning. A light source, usually a hollow cathode lamp, emits light at a wavelength specific to the element under analysis. As the light passes through the graphite furnace, free atoms within absorb a portion of it at the specific wavelength. The detector then measures the amount of light absorbed, which is directly proportional to the element’s concentration in the sample. ETAAS offers significantly higher sensitivity than FAAS, making it the preferred method for quantifying beryllium at low levels across various sample types. However, certain limitations still exist, and to address these, adjustments such as pyrolytically coated graphite tubes [13] or metal atomizers [14] have been implemented. These modifications improve signal reproducibility and minimize memory effects compared to uncoated graphite tubes [15]. Using the Stabilized Temperature Platform Furnace (STPF) concept, which incorporates a suitable chemical modifier, no undesirable effects were observed in the quantification of beryllium in both synthetic mixtures and real-world samples [13]. Chemical modification may involve various agents, including Mg(NO_3_)_2_ [16,17,18,19], La(NO_3_)_3_ [20], Pd(NO_3_)_2_ [21], ZrOCl_2_ [22], Al(NO_3_)_3_ [23], acetylacetone in ammonium acetate buffer [24], a mixture of ammonium phosphomolybdate and ascorbic acid [25], and lutetium [26,27]. These chemical modifiers primarily serve to stabilize beryllium, enabling higher thermal decomposition (ashing) temperatures without analyte loss, reducing matrix interferences, and enhancing sensitivity.

In the analysis of natural waters and wastewater, Mg(NO_3_)_2_ allowed for an ashing temperature of 1300 °C, improving both sensitivity and accuracy compared to measurements conducted without a chemical modifier [16]. For biological samples, specifically blood and blood serum, Mg(NO_3_)_2_ was effective only in blood analysis, where it increased sensitivity. In this case, an ashing temperature was set to 800 °C [18]. La(NO_3_)_3_ was employed in the determination of beryllium in Chinese yellow rice samples after its separation and concentration on a TiO_2_@SiO_2_ flexible nanofiber membrane. Reliable results were achieved with an ashing temperature of 1100 °C [20]. The highest thermal decomposition temperature, 1500 °C, was used with Pd(NO_3_)_2_. It is important to note that this measurement was conducted using graphite tubes with inserted zirconium-coated platforms, which served as a permanent chemical modifier [21]. Pd(NO_3_)_2_ was also one of the three chemical modifiers tested by Shimizu et al. [28], along with Mg(NO_3_)_2_ and Ni(NO_3_)_2_. Among these, Pd(NO_3_)_2_ provided the highest sensitivity for beryllium. At an ashing temperature of 1000 °C, its use enhanced sensitivity by 3.7 times compared to measurements without a chemical modifier. When zirconium was used as a chemical modifier, pyrolysis was performed in two steps at 900 °C and 1000 °C. Additionally, using a tungsten platform in a pyrolytically coated graphite tube enhanced sensitivity by 2.5 times compared to a pyrolytic graphite platform [22]. 

**Figure 1:**
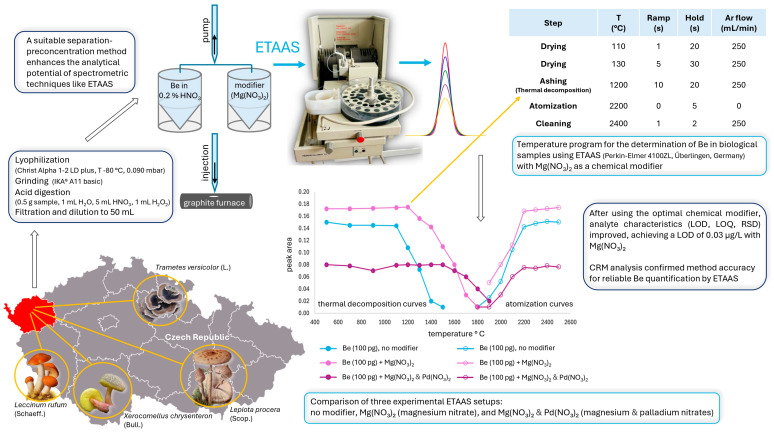
Optimization of the temperature program for trace beryllium quantification in fungi using electrothermal atomic absorption spectrometry (ETAAS). Thermal decomposition and atomization curves were compared for three setups: no modifier, Mg(NO_3_)_2_, and Mg(NO_3_)_2_ + Pd(NO_3_)_2_. The optimal thermal decomposition temperatures were 1100 °C (no modifier), 1200 °C (Mg(NO_3_)_2_), and 1500 °C (Mg(NO_3_)_2_ + Pd(NO_3_)_2_), with a consistent atomization temperature of 2200 °C. The lowest limit of detection (LOD) of 0.03 µg/L and the results of the analysis using certified reference material (CRM) confirmed Mg(NO_3_)_2_ as the most suitable modifier for reliable beryllium quantification.

Based on our experimental work, we designed a figure illustrating the optimization of the temperature program for trace beryllium quantification in fungi using ETAAS. It visually outlines the process from sample collection to temperature optimization and measurement, demonstrating the use of Mg(NO_3_)_2_ and Pd(NO_3_)_2_ as chemical modifiers (Figure 1).

Inductively coupled plasma optical emission spectrometry (ICP-OES) detects the light emitted by atoms and ions excited in a high-temperature plasma, typically ranging from 6000 to 10,000 K. When these excited species return to lower energy states, they emit light at wavelengths specific to each element. This emitted light is collected and separated by a spectrometer, with the intensity at each wavelength measured by a detector. The intensity of the emitted light is directly proportional to the concentration of each element in the sample, enabling accurate quantification. ICP-OES can simultaneously analyze multiple elements with relatively high sensitivity, making it a widely adopted method for analyzing a variety of samples. Its ability to handle multiple elements at once makes it particularly valuable in laboratories that need to monitor numerous samples and elements in parallel. For beryllium, ICP-OES is effective for quantifying moderate to high concentrations [29], or low levels when coupled with an appropriate separation and preconcentration procedure [30,31,32].

Inductively coupled plasma mass spectrometry (ICP-MS) is a powerful technique used to detect and quantify trace elements and isotopes in a sample. In ICP-MS, a liquid sample is first converted into an aerosol and introduced into a high-temperature plasma (typically around 6000–10,000 K), where it is atomized and ionized. The resulting ions are extracted into a mass spectrometer, where they are separated based on their mass-to-charge ratio (*m*/*z*). These ions are then detected, and their intensities are measured to determine the element concentration in the sample. ICP-MS is known for its high sensitivity and multi-elemental capability, allowing it to detect elements at extremely low concentrations. Among spectrometric techniques, it is one of the most sensitive for quantifying beryllium. ICP-MS can detect ultra-trace levels of beryllium across a variety of matrices, making it ideal for environmental [33,34], biological [35,36,37,38,39,40,41], and occupational health monitoring [42].

While ICP-MS offers excellent detection capabilities, it is highly susceptible to matrix effects (e.g., interferences caused by sample components, space-charge effects, ion suppression, and changes in plasma conditions), which can limit its performance [43]. Beryllium, with its low mass, is particularly prone to being affected by space-charge effects, where heavier elements in the sample displace lighter ones from the ion beam, resulting in reduced sensitivity [44]. Matrix effects are more pronounced in inorganic samples (e.g., geological and environmental samples with high salt content) due to the presence of high concentrations of dissolved solids, which interfere with ionization efficiency and transmission in the mass spectrometer. In contrast, biological samples (e.g., blood, urine, and tissues) typically contain lower levels of dissolved salts, making matrix effects generally less severe. Additionally, modern ICP-MS instruments can be specifically tuned for low-mass measurements (e.g., Be at *m*/*z* = 9), where *m*/*z* refers to the mass-to-charge ratio of Be ions detected in ICP-MS. Since beryllium’s most abundant isotope is ^9^Be (with a mass of approximately 9 amu) and it typically forms a singly charged ion (Be^+^, with *z* = 1), its *m*/*z* ratio is 9/1 = 9. Optimizing ICP-MS conditions for low-mass measurements helps minimize space-charge effects. However, this does not mean that biological samples are completely free from matrix effects—proteins, lipids, and other organic components can still contribute to ionization suppression, though to a lesser extent than in highly mineralized samples. The severity of matrix effects depends on sample composition, preparation steps, and the tuning parameters of the ICP-MS instrument. Therefore, developing effective separation and preconcentration techniques is essential for the reliable quantification of beryllium in complex matrices, especially those with high salt concentrations [45].

X-ray fluorescence (XRF) spectrometry is a technique used to determine the elemental composition of materials. In XRF, a sample is exposed to high-energy X-rays, which cause the atoms within the material to emit secondary X-rays (fluorescent X-rays) that are characteristic of the elements present. These emitted X-rays are then detected, and their energies are used to identify the elements, while their intensities provide information about the concentration of each element in the sample. XRF is a non-destructive method widely used for the rapid screening of various elements in solid samples, such as soils, dust, or metal alloys. It is typically applied to determine elemental composition at ppm or percent levels but is not suitable for trace or ultra-trace analysis. Although XRF is less sensitive than some other analytical techniques, it remains valuable for quick, on-site analysis. Moreover, XRF encounters significant challenges when detecting elements with an atomic number of 9 or lower. These limitations become increasingly pronounced as the atomic number decreases, making direct quantification of beryllium (atomic number 4) nearly impossible. To overcome these issues, indirect methods for beryllium quantification with XRF have been proposed [46]. Alternatively, employing specialized instrumentation and/or using specific sample preparation techniques can improve the accuracy of beryllium quantification.

Laser-induced breakdown spectroscopy (LIBS) is an analytical technique used to determine the elemental composition of materials by focusing a high-powered pulsed laser at a target (material to be analyzed), generating a plasma from the target material within the surrounding medium (ambient air, a vacuum, or some other controlled atmosphere), enabling the spectral analysis of the emitted light. The resulting plasma, reaching temperatures between 10,000 and 25,000 K in less than 10 microseconds, is hot enough to break down molecules into atoms or ions and to excite electrons in both neutral atoms and ions from their ground state to higher energy levels. As these excited species return to their ground states, they emit characteristic radiation across a broad spectral range, from the infrared (IR) to the X-ray region. The identification of atoms or ions within the sample is achieved using well-established emission lines, and elemental concentrations are determined by measuring the intensity of these lines. LIBS is a rapid and versatile technique that requires minimal sample preparation and is capable of analyzing various materials, though it faces challenges like matrix effects and spectral overlap. For beryllium quantification at higher concentration levels, LIBS commonly and effectively uses the ionic Be II emission doublet lines at 313.04 and 313.11 nm [47]. However, determining beryllium concentrations in the range of 0.1–5 wt% with this doublet is challenging due to its resonance nature, which leads to strong self-absorption [48]. Several studies have confirmed that at higher beryllium concentrations, reduced sensitivity due to self-absorption in the plasma occurs [49,50,51], highlighting the need for careful calibration and spectral analysis. One solution to this problem is to use LIBS to detect the BeO diatomic molecular emission, a technique known as molecular LIBS (MLIBS) [51,52].

Fluorescence spectroscopy (FS) is an analytical technique used to examine the fluorescent properties of substances by measuring the light they emit after absorbing light or other forms of electromagnetic radiation. During this process, a sample is exposed to light, typically in the ultraviolet or visible range, which excites the electrons in the molecules to a higher energy state. As the electrons relax, they release energy and eventually return to their ground state by emitting light with a longer wavelength, known as fluorescence. This emitted light is detected and analyzed, providing valuable information about the sample’s composition, concentration, and molecular environment. When FS is used for quantifying an element, it involves the formation of a fluorescent complex with the element of interest, typically by reacting it with specific reagents. The fluorescence intensity of the resulting complex is then measured. For beryllium ions, the reagent is usually a fluorescent dye or molecule that forms a stable complex with beryllium, exhibiting fluorescence. Common reagents include hydroxybenzoquinoline sulfonate (HBQS) [53,54,55], beryllon II [56], and morin [57,58], all of which form highly fluorescent complexes with beryllium ions. Although less common, this method is used in specialized applications.

UV-Vis spectrophotometry (UV-Vis) measures light absorption by a substance at specific wavelengths within the ultraviolet and visible regions of the electromagnetic spectrum. A light source emits light across these regions, and a monochromator selects particular wavelengths to pass through the sample, which is usually contained in a cuvette in liquid form. As light interacts with the sample, certain wavelengths are absorbed depending on the concentration of the absorbing molecules. The remaining light passes through the sample and is detected by a photodetector, which measures the intensity of the transmitted light. Absorbance is calculated by comparing the intensity of incident light to that of transmitted light. This absorbance value is then plotted against wavelength to generate an absorption spectrum, which reveals information about the sample’s molecular structure and concentration based on the wavelengths at which absorption occurs.

The quantification of beryllium by UV-Vis spectrophotometry requires the formation of colored complexes using specific reagents, which are then analyzed by measuring absorbance in the UV-Vis range. The development and application of spectrophotometric techniques for beryllium analysis have been extensively researched over several decades, resulting in the identification of numerous organic reagents capable of forming vividly colored beryllium complexes with high molar absorptivities. Important photometric reagents include hydroxy acids, chromotropic acids, β-diketones, azo dyes, and derivatives of hydroxyquinone and triphenylmethane. Among the spectrophotometric reagents suitable for beryllium analysis, chrome azurol S (CAS) is one of the most widely used [59,60,61,62,63]. Other important reagents worth mentioning are anthralin [64] and hematoxylin [65]. This method is particularly suitable for simple matrices with high beryllium concentrations. Beryllium’s tendency to form stable complexes affects its quantification in UV-Vis and FS, where chelation with organic reagents is necessary but adds complexity to the analysis. In ICP-MS, ICP-OES, and FAAS, beryllium complexation with strong ligands can lead to signal suppression due to incomplete dissociation during atomization or ionization.

Among the techniques mentioned above, only ICP-MS is suitable for quantifying very low levels of beryllium in natural samples. Most other methods require integration with an appropriate separation/preconcentration procedure to reliably measure ultra-trace concentrations of this element. Later in the text, we will discuss various effective separation techniques combined with spectrophotometry, flame atomic absorption spectrometry, electrothermal atomic absorption spectrometry, and inductively coupled plasma optical emission spectrometry in more detail.

### Comparison of Spectroanalytical Techniques for Beryllium Quantification

When selecting an analytical technique for beryllium quantification, factors such as sensitivity, specificity, susceptibility to matrix interferences, and practical considerations like cost and sample preparation must be weighed (Table 1).

FAAS and ETAAS are generally more affordable compared to ICP-based techniques, with FAAS being the simpler option. FAAS is commonly used for routine analysis due to its straightforward operation, minimal expertise requirements, and lower cost. However, it is limited to higher concentration levels (typically in the mg/L range) and is more susceptible to matrix interferences such as spectral overlaps or chemical interactions with co-existing ions (e.g., Al^3+^, Fe^3+^). On the other hand, the interferences in FAAS are typically more predictable and less complex compared to those in some other methods, allowing easier correction via background subtraction or matrix modifiers [66]. In contrast, ETAAS offers higher sensitivity, enabling the detection of beryllium at trace concentrations (ng/L to µg/L levels), but it involves intricate sample preparation and optimization. Beryllium forms refractory compounds, such as the stable oxide BeO, which require high atomization temperatures for complete dissociation into free atoms. Inadequate heating during the atomization step fails to break Be-O bonds, reducing sensitivity, while excessive temperatures (overheating) during the ashing step risk the premature volatilization of beryllium, leading to analyte loss [67]. ETAAS thus relies on precise temperature programming across three critical phases to effectively remove the matrix and atomize the analyte. First, *drying* eliminates solvents without splattering. Next, *ashing* removes organic and inorganic matrix components at controlled temperatures (<1500 °C for Be [e.g., 16,20–24,28]) to prevent analyte loss (Be vaporizing before measurement). Finally, the *atomization* involves rapid heating to ~2300–2900 °C [16,22,24,68,69], ensuring the release of beryllium atoms for accurate quantification. Each phase is crucial for maintaining sensitivity and precision in ETAAS analysis. Complex matrices, such as environmental or biological samples, may require optimized thermal profiles to prevent the co-volatilization of beryllium with interfering substances. Additionally, these samples may require chemical modifiers (e.g., Mg(NO_3_)_2_, [16,18], Pd(NO_3_)_2_ [21,28]) to stabilize beryllium during ashing. ETAAS demands specialized graphite furnaces, meticulous temperature control, and operator expertise to balance these factors, making it more complex and costly than FAAS.

For applications requiring higher sensitivity and multi-element analysis, ICP-based techniques are more suitable. ICP-OES is suitable for routine analysis, particularly for elements like beryllium. It offers reliable sensitivity at the ppb level and supports high-throughput analysis, making it ideal for regular monitoring, especially when analyzing multiple elements simultaneously. However, matrix interferences may require optimization to ensure accurate results, depending on the sample’s complexity. ICP-MS offers the highest sensitivity, detecting beryllium at ultra-trace levels with excellent specificity. Isobaric interferences for beryllium in ICP-MS analysis are generally low due to its monoisotopic nature (^9^Be) and low *m*/*z*, which avoids common polyatomic overlaps prevalent at higher masses. However, quantification challenges arise from its high first ionization potential (9.3 eV), resulting in only 70–75% of beryllium being ionized in a standard argon plasma. Additionally, being a very light element (9 amu), beryllium analysis is further complicated by space-charge effects within the ion beam. These shortcomings can be addressed by increasing the plasma power and optimizing the ion optics specifically for beryllium analysis [70,71]. While high matrix complexity could theoretically introduce rare polyatomic species, modern instrumentation effectively mitigates such interferences, leaving ionization efficiency and ion beam dynamics as the primary analytical constraints.

These limitations in solution-based ICP-MS have led to the development of alternative approaches, such as laser ablation ICP-MS (LA-ICP-MS), which bypasses the need for sample digestion and enables direct solid sample analysis. LA-ICP-MS offers high sensitivity, low detection limits, and the ability to detect elements lighter than sodium, including beryllium [72]. This technology enables in situ, real-time, rapid, and simultaneous multi-element detection along with isotopic ratio analysis [73]. It is widely used in gemological laboratories for identifying beryllium in gemstones [72].

To further improve the precision of ^9^Be analysis in geological reference materials, Sproson et al. [33] introduced a new analytical procedure using high-resolution HR-ICP-MS. Their approach significantly reduced the error associated with ^9^Be reactive phase measurements, achieving a precision range of 0.1 to 1.4%, whereas previous methods, such as AAS, ICP-OES, and ICP-MS, typically achieved a precision of 2 to 5%. This advancement has important implications for Earth Sciences, as it minimizes uncertainty in ^10^Be/^9^Be ratios and allows for a reduced sample size or leaching solution strength without compromising accuracy.

Spectroscopic techniques like FS and UV-Vis are lower-cost alternatives for beryllium quantification but come with significant limitations, including high susceptibility to matrix interferences and generally lower specificity. FS can achieve good sensitivity in the ppb to ppm range under certain conditions, particularly when fluorescence-based derivatization is applied. However, its effectiveness is highly dependent on the chemical environment [74,75,76]. Since beryllium itself is not fluorescent, its detection via FS requires complexation (a form of derivatization) with suitable organic reagents (e.g., morin, beryllon II, or hydroxyquinoline derivatives), which form highly fluorescent compounds. These complexes enhance detectability by significantly increasing fluorescence intensity, allowing detection at very low concentrations in optimal conditions. This is because fluorescence detection relies on emitted light, which, under ideal conditions, can provide a stronger [77,78,79] and more selective [80] signal than the absorbed light in techniques like FAAS. Some studies report detection limits in the ng/L to sub-ppb (<1 µg/L) range [55,81], making FS competitive with other trace beryllium detection techniques. Unlike absorption-based methods such as UV-Vis, FS benefits from a lower background signal (interference), which can improve sensitivity when ideal conditions are met. However, FS is highly prone to matrix interferences, as real-world samples (e.g., environmental, biological, or industrial) often contain substances that (1) quench fluorescence by interacting with sample components and reducing signal intensity, (2) scatter or absorb excitation/emission light, leading to signal loss or distortion, and/or (3) compete with Be for complexation, reducing the formation of the desired fluorescent Be complex. FS also typically has lower specificity compared to techniques like ICP-MS or ETAAS. The fluorescence response depends on the reagent used, and some organic ligands (e.g., morin and beryllon II) can form complexes with other metal ions [82,83], potentially leading to false positives or requiring additional masking agents for selective beryllium detection.

UV-Vis, while simple and inexpensive, lacks the sensitivity and specificity needed for reliable beryllium quantification, particularly at low concentrations. Nonetheless, some colorimetric methods using UV-Vis spectrophotometry can achieve detection of beryllium in the low ppb range. For example, Taylor and Sauer [84] described a colorimetric technique capable of detecting beryllium on surfaces at concentrations as low as 0.2 micrograms per 100 cm^2^, which is equivalent to 2 ppb. This demonstrates that, with appropriate reagents and conditions, UV-Vis spectrophotometry can achieve the sensitivity required for low-level beryllium detection. Since beryllium itself does not absorb significantly in the UV-Vis range, complexation with organic ligands (e.g., CAS [63], anthralin [64]) is necessary to form chromogenic complexes that create detectable absorption changes, thereby enhancing its detectability.

For solid sample analysis, XRF and LIBS provide the advantage of minimal sample preparation and rapid analysis. XRF is non-destructive and offers moderate sensitivity, though it is less effective for detecting light elements like beryllium, due to their weak fluorescence signals. Beryllium’s X-ray emissions are either too weak or absorbed by air or the detector window [85,86]. LIBS, on the other hand, is faster and offers superior spatial resolution, but Be’s low emission intensity (due to its low atomic number) can limit sensitivity compared to heavier elements. According to published reports, LIBS sensitivity for light elements such as beryllium can be accurate down to the 1–10 ppm level [87]. Moreover, matrix effects in LIBS can further complicate ultra-trace detection, making it less reliable for applications that require high precision. Despite these limitations, both techniques remain valuable tools for the elemental analysis of solid samples, particularly when used complementarily.

Additionally, LA-ICP-MS, is also highly suitable for analyzing solid materials and offers greater analytical precision and significantly lower detection limits for all trace elements, including beryllium, compared to XRF and LIBS. While XRF detection limits range from ~1000 ppm for light elements to ~1 ppm for heavy elements, LA-ICP-MS typically achieves sub-ppm levels for most elements, reaching ppb for many heavy elements. Furthermore, LA-ICP-MS can detect light elements like beryllium, which XRF struggles with due to low-energy X-ray emission [72]. Another key advantage is its ability to analyze solid samples directly, making it useful for certain applications.

Ultimately, the choice of technique depends on the required detection limits, sample type, and available resources. While ICP-MS is the most powerful for ultra-trace level detection, methods like ETAAS or ICP-OES may be more practical in many laboratory settings. XRF, LIBS, and LA-ICP-MS offer advantages for direct solid analysis, while FS and UV-Vis remain useful for specific applications despite their limitations.

## 3. Extraction Techniques for the Separation and Preconcentration of Beryllium

Although higher concentrations of beryllium are commonly found in environments impacted by anthropogenic pollutants, such as those resulting from fossil fuel combustion for power generation and transport, or industrial processes like mining and smelting [3,34,88,89], many natural samples still contain only trace or ultra-trace amounts of this element. Given that even very low levels of beryllium can be toxic to living organisms, accurate quantification is essential for all types of samples.

Even though spectrometric methods offer relatively high sensitivity and selectivity, ultra-trace analysis remains a significant challenge. The two main issues are: (1) the analyte’s concentration in the sample is often extremely low, complicating accurate measurement, and (2) the sample may contain numerous other components at much higher concentrations than the analyte, leading to potential interference. To address these challenges, employing an effective separation technique that separates and preconcentrates the analyte of interest is a practical solution.

Among separation techniques, extraction is one of the most widely used ones. In the analysis of liquid samples, extraction can occur either between a solid and a liquid phase or between two immiscible liquid phases, depending on the preferred technique. When the analyte is captured on a solid material, the process is referred to as solid-phase extraction (SPE). Alternatively, liquid-liquid extraction (LLE) involves the partitioning of the analyte between two unmixable liquid phases. Since their introduction, both extraction techniques have undergone numerous modifications, many of which align with the principles of green chemistry. The following discussion will explore some of the key modifications, both historical and recent.

### 3.1. Liquid-Liquid Extraction

Liquid-liquid extraction (LLE), also known as solvent extraction, operates on the principle that an analyte can partition itself between two immiscible solvents: typically, water (an aqueous phase) and an organic solvent (an organic phase) in a specific ratio. Initially, this technique involved the use of large volumes of hazardous organic solvents and sample solutions, often making the process both time-consuming and wasteful. Such practices generated substantial amounts of hazardous waste, which was not in line with the principles of green chemistry [90].

Modern analytical chemistry emphasizes eliminating hazardous solvents entirely and focuses on simplifying and miniaturizing sample preparation procedures. This approach reduces the consumption of samples, solvents, and reagents, leading to a significant decrease in laboratory waste. In this context, several modifications of traditional LLE have been developed, including single-drop microextraction (SDME), cloud-point extraction (CPE), dispersive liquid-liquid microextraction (DLLME), solidification of a floating organic droplet in dispersive liquid-liquid microextraction (SFO-DLLME), vortex-assisted liquid-liquid microextraction (VALLME), and others [91].

An earlier method for isolating beryllium from coal fly ash discharged by thermal power plants utilized a β-diketone liquid chelating exchange agent [92]. Among the various organic solvents tested, such as cyclohexane, isoamyl acetate, methyl isobutyl ketone, chloroform, carbon dioxide, and 1,2-dichloroethane, cyclohexane at pH 9.5 was the most effective for quantitatively extracting the Be-β-diketone complex. The extracted beryllium was then re-extracted into a 2 M HCl solution for quantification by ETAAS. Although relatively high concentrations of beryllium in the coal fly ash samples indicated that direct quantification might be feasible, this approach was not entirely reliable due to interference from other ions present in the samples. In the absence of the separation procedure, some measurements showed beryllium concentrations to be more than 300% higher than expected, likely due to interference from other ions. After applying the separation procedure, beryllium concentrations in the coal fly ash samples were found to range between 0.92 and 4.69 µg/g.

A liquid-liquid extraction paired with ICP-OES, where Be^2+^ ions were complexed with acetylacetone and extracted into p-xylene, achieved an impressive enhancement factor of 1300 [30]. The high flow rate ratio between the sample solution and p-xylene led to an enrichment factor of 75. Additionally, p-xylene (used as an organic solvent) contributed an extra enhancement of 10–20, making the observed factor of 1300 reasonable. The method achieved a limit of detection (LOD) of 8.0 ng/L for beryllium, a substantial improvement compared to the 3.1 µg/L LOD obtained via the conventional nebulization of an aqueous solution without liquid-liquid extraction. However, it is worth noting that this method was tested only on model solutions, with no real-world sample analysis conducted.

For the separation and preconcentration of ultra-trace levels of beryllium from effluents and natural water samples, two surfactant-assisted dispersive liquid-liquid microextraction (SA-DLLME) methods were developed [21,93]. In the first, cetylpyridinium ammonium bromide (CPAB), a cationic surfactant, served as both the ion-pairing and dispersing agent. The positively charged part of the CPAB molecule formed hydrophobic ion-pair complexes with the negatively charged beryllium-oxalate species, which were then extracted into chloroform [93]. In the second, dioctyl sodium sulfosuccinate (AOT), an anionic hydrophobic double-tailed surfactant, acted as the dispersing agent, forming vesicles that captured beryllium in the presence of salicylic acid, which were subsequently extracted into a small volume of chloroform [21]. For beryllium quantification, ETAAS was used. Both methods achieved a preconcentration factor of 25. Beryllium concentrations in drinking water and groundwater samples were below the LOD of 1 ng/L, while concentrations in effluent samples ranged from 9 to 92 µg/L. A highly sensitive vesicle-mediated dispersive liquid-liquid microextraction (VM-DLLME) procedure was developed for the quantification of beryllium at parts-per-quadrillion levels in seawater, air filter, and coal fly ash samples [94]. In this method, the anionic surfactant dioctyl sulfosuccinate (DOSS) serves as the dispersing agent, forming vesicle aggregates. After complexation with acetylacetonate, beryllium interacts with the hydrophobic solubilizing sites of the vesicles, enabling its extraction from the bulk aqueous phase into a small chloroform phase. The beryllium in chloroform is then back-extracted into diluted nitric acid, and this solution is used for beryllium quantification by ETAAS. Under optimized conditions, the method achieved an LOD of 0.01 ng/L for seawater, 0.15 ng/g for air filters, and 1.5 ng/g for coal fly ash samples.

### 3.2. Cloud-Point Extraction

Cloud-point extraction (CPE) is a specialized form of LLE that employs non-toxic surfactants as the extraction medium. It relies on non-ionic or zwitterionic surfactants, which form micelles in aqueous solutions when their concentrations exceed the critical micelle concentration (CMC). These micelles are spherical structures in which the hydrophobic tails of the surfactant molecules cluster together in the center, creating a non-polar core, while the hydrophilic heads remain oriented outward, interacting with the surrounding water. Micelle formation and the resulting turbidity occur spontaneously when the solution is heated to a specific temperature, referred to as the cloud-point temperature (CPT). Beyond this temperature, the initially uniform micellar solution separates into two distinct phases: a small-volume, surfactant-rich phase predominantly consisting of surfactant molecules, and a diluted aqueous phase where the surfactant concentration remains near the CMC.

In elemental analysis, the process typically begins with the addition of a complexing agent that forms hydrophobic complexes with the target element. These complexes are captured within the hydrophobic cores of the micelles and subsequently separate into the surfactant-rich phase, making their extraction easier. This process enhances both the extraction and concentration of the analyte, significantly improving the efficiency of the overall procedure [95,96].

Based on the information provided, it is evident that surfactants and complexing agents are crucial for developing effective extraction procedures. Among non-ionic surfactants, Triton X-114 (polyoxyethylene-7.5-octylphenoxy ether) stands out as the most commonly used agent in CPE procedures. Notably, over 80% of studies employing CPE for the separation, preconcentration, and speciation of inorganic analytes have utilized Triton X-114 [97]. In the context of beryllium analysis, Triton X-114 has been applied either on its own [17,32,98,99] or in combination with other surfactants such as cetylpyridinium chloride (CPC) [100,101] or cetyltrimethylammonium bromide (CTAB) [61]. When surfactants are combined, commonly by mixing non-ionic surfactants with ionic or zwitterionic ones, the procedure is referred to as mixed-micelle CPE (MM-CPE). This approach leads to the formation of mixed micelles with unique properties and often results in modifications to key parameters such as CMC and CPT.

In addition to surfactants, complexing agents play a vital role in extraction procedures. For beryllium analysis, a variety of complexing agents have been utilized, including chrome azurol S [60,61,98], 1,8-dihydroxyanthrone [64,101], 4-sulfamoylphenyl dithiocarbamate [99], and cupferron [17]. Recently, there has been a shift toward using nanomaterials as sorbents in various extraction procedures, including CPE. These materials facilitate the transport of analytes into the surfactant-rich phase, thereby minimizing the reliance on complexing agents. For instance, studies have demonstrated that beryllium can adsorb onto graphene oxide (GO) nanosheets, which are then preconcentrated using CPE [32]. This innovation represents a promising direction for improving extraction efficiency in analytical procedures.

Among spectrometric techniques, UV-Vis [60,61,64,98], ICP-OES [32,99,101], and ETAAS [17] have been employed for beryllium quantification following its separation and preconcentration by CPE. All studies that applied the CPE procedure reported an improved LOD. This parameter varies considerably across methods: for UV-Vis combined with CPE, the LOD ranges from 0.05 to 0.98 µg/L; for ICP-OES combined with CPE, it ranges from 0.001 to 0.005 µg/L; and for ETAAS combined with CPE, the reported LOD is 0.02 µg/L. The applicability of these methods was demonstrated through the analysis of real-world samples. Natural water samples of various origins and compositions were analyzed in all of the studies mentioned, with the exception of one that analyzed boron samples, including boron waste, boron ore, and processed boron ore [99]. Beryllium, quantified in several seawater samples, had reported mean concentrations of 0.13 µg/L [100], 4.93 µg/L and 5.61 µg/L [60], and 2.38 µg/L [101]. Additionally, wastewater samples were examined, revealing mean beryllium concentrations of 10.0 µg/L in influent and 1.00 µg/L in effluent samples [32]. The beryllium level in specific samples, such as boron, was below the LOD [99].

### 3.3. Solid-Phase Extraction

Solid-phase extraction (SPE) relies on a solid material to effectively retain the analyte of interest. A liquid sample is passed through a (mini)column, capillary, disk, cartridge, or syringe containing this material. The solid phase has a surface with specific chemical and/or physical properties that interact with the analyte based on its characteristics (e.g., polarity, charge, or hydrophobicity), allowing selective retention while other components pass through. However, column-based SPE often presents issues such as sorbent leaching, channeling, and cartridge clogging, which may extend the procedure beyond the desired timeframe. An alternative approach involves using the solid phase as a sorbent in a dispersion mode, characteristic of dispersive solid-phase extraction (DSPE) [102].

In DSPE, a small amount (typically tens of milligrams) of the solid phase is added directly to the liquid sample, and the mixture is stirred for a few minutes to promote analyte adsorption. Phase separation is then performed, usually by centrifugation, to remove the aqueous phase while retaining the solid phase with the adsorbed analyte. Depending on the detection technique used for further analysis, the next steps may include: (1) eluting the analyte from the solid phase with a small volume of a suitable eluent (typically hundreds of microliters to a few milliliters), (2) dissolving the solid phase containing the adsorbed analyte with a small volume of a suitable reagent, ensuring that the sorbent dissolves easily and does not introduce interferences, (3) drying the solid phase with the adsorbed analyte, followed by direct analysis (e.g., when using energy-dispersive X-ray fluorescence spectrometry (EDXRF) as a detection method), or (4) preparing a slurry by adding a small amount of a suitable reagent and directly analyzing the sample (e.g., for ETAAS in slurry sampling mode) [102].

A variety of sorbents for beryllium have been reported in the literature, including silica-based sorbents (both unmodified and modified), carbon-based sorbents (such as activated carbon, functionalized graphene, and graphene oxide), magnetic sorbents (e.g., MIIPs and magnetic MOFs), as well as specialized sorbents like oasis cartridges, strong-base anion-exchange resins, human hair, and TiO_2_@SiO_2_ flexible nanofiber membranes. These sorbents will be discussed in detail in the following sections.

#### 3.3.1. Silica-Based Sorbents

Silica-based sorbents, primarily composed of silica (silicon dioxide, SiO_2_), are materials widely used for adsorption in various separation and purification processes due to their excellent adsorptive properties, large surface area, and chemical stability. These materials are available in both unmodified and modified forms. Among the modified variants, octadecyl silica sorbents (C18) are the most common, with ‘C18’ referring to the length of the hydrocarbon chains chemically bonded to the silica surface. Other modifications, such as octadecyl silica gel treated with aluminon, octadecyl cartridges modified with quinalizarin, or silica gel bonded with benzenesulfonic acid, will be discussed later in the text.

As mentioned earlier, ICP-MS is known for its strong detection capabilities but can be limited by its sensitivity to matrix effects. Due to its low mass, beryllium is particularly vulnerable to space-charge effect (a specific type of matrix effect), where heavier elements can interfere with the ion beam, reducing sensitivity. Therefore, separation and preconcentration are essential for reliable quantification of beryllium in complex matrices, particularly those with high inorganic content, when using ICP-MS.

In seawater analysis, an effective approach involves using SPE technique with commercially available silica gel packed in a polypropylene column [45]. As seawater passes through the silica gel column, interfering matrix ions are removed by eluting with an EDTA solution, while beryllium remains bound to the silica gel. The beryllium is then quantitatively recovered by elution with HNO_3_. This method was applied to analyze the vertical profile of beryllium in the eastern North Pacific Ocean, yielding interesting results. The beryllium concentration profile remained steady at depths of 10 to 200 m, with levels around 7 pmol/kg. It then rose with increasing depth from 200 to 3500 m, reaching approximately 30 pmol/kg before stabilizing between 3500 and 5000 m.

Among the elements quantified by ETAAS, beryllium has one of the lowest LODs. However, various preconcentration procedures have been developed to further improve its detection. One approach involves SPE using a custom-made C18 cartridge for the preconcentration of beryllium from hair samples [68] and marine organisms, such as fish, shrimp, octopus, lobster, oyster, and algae [69]. In both studies, beryllium was complexed with acetylacetone (acac) in the presence of an acetate buffer solution (pH 6.0). The procedures involved sample decomposition using a mixture of HNO_3_ and H_2_O_2_ under microwave irradiation, followed by the addition of acetylacetone to form the Be(acac)_2_ complex. This complex was retained on the C18 cartridge and subsequently eluted with methanol. The resulting methanolic solution was then introduced into a graphite furnace for ETAAS analysis. The reliability of the method was confirmed by analyzing certified reference materials (CRMs) of biological matrices and/or by applying the standard addition method. Beryllium concentrations in hair samples collected from healthy individuals ranged from 6.0 to 7.6 ng/g [68], while concentrations in marine organisms ranged from 7.1 to 24.7 ng/g [69].

A similar approach was applied to natural water samples (groundwater and spring water), utilizing a commercially available C18 cartridge (Sep-Pak C18) to capture the Be(acac)_2_ complex, which was then eluted with methanol [103]. In this study, measurements were conducted using uncoated graphite cuvettes (tubes). Typically, pyrolytically coated tubes are preferred due to their lower porosity and greater resistance to high temperatures. Furthermore, the results were evaluated based on absorbance peak heights, which is a rather non-standard practice. Peak heights are generally not recommended for evaluation, as they are largely unreproducible and strongly depend on the quality of the graphite tube surface. Beryllium concentrations in the analyzed natural water samples were below the LOD (2.3 ng/L).

In another study, the adsorption of beryllium onto the surface of silica fibers from an alkaline medium (pH 9.5) was optimized for its separation and preconcentration from lake and seawater [104]. The results indicated that the adsorbed beryllium species were either (-SiO)Be(OH)_2^−^_ or (-SiO)_2_Be(OH)_2_^2^−^^. These species were separated on three-layer filters containing silica fibers, and desorption was achieved using 0.5 M HCl prior to ETAAS measurements. After optimization, an impressive preconcentration factor of 300 was achieved. Despite this, beryllium concentrations in polluted lake water and unpolluted seawater samples were below the LOD (0.3 ng/L).

Farahmand [11] employed octadecyl silica gel modified with aurin tricarboxylic acid (aluminon) to separate and preconcentrate beryllium from tap water samples. The aluminon forms a complex with Be^2+^ ions. After passing the sample containing Be^2+^ through the cartridge, beryllium was extracted by rinsing with 0.1 M HNO_3_. The amount of beryllium was then measured using FAAS. Following optimization, an enrichment factor of 330 was achieved, resulting in an LOD of 0.1 µg/L. However, the concentration of beryllium in the tap water samples was found to be below this detection limit.

An octadecyl silica cartridge modified with quinalizarin as a chelating agent was successfully used to extract beryllium from both simple matrices, such as tap and well water, and more complex matrices, such as Cu-Be alloy samples [105]. At a pH of 6–6.6, beryllium in the form of Be^2+^ was retained on the modified cartridge and then eluted with 0.5 M HNO_3_ for quantification by FAAS. This method achieved an enrichment factor of 200 and an LOD of 0.2 µg/L. While beryllium concentrations in water samples were below the LOD, alloy samples contained beryllium at concentrations of 1.55% and 1.85%. Despite these relatively high percentages, the presence of other metals in significantly greater amounts posed a challenge for reliably quantifying beryllium even at concentrations this high. The use of an effective separation process, however, enhanced selectivity, demonstrating the method’s applicability to complex matrices.

A functionalized nanosized mesoporous silica (MCM-41), modified with 2,4-dihydroxybenzaldehyde (4-OHsal), was used as an efficient sorbent for the extraction of Be^2+^ ions from aqueous solutions, followed by ICP-OES quantification [31]. SPE was carried out in batch mode, where 10 mg of the sorbent was added to a test tube containing the sample solution, followed by manual shaking and centrifugation. The aqueous phase was then decanted, beryllium was desorbed from the sorbent with 1.0 M HNO_3_ and quantified by ICP-OES. This method was successfully applied for preconcentrating beryllium in water samples from various sources. Under optimal conditions, the method achieved an LOD of 0.3 ng/L. While beryllium concentrations in tap water were below the LOD, well water contained 0.028 µg/L and seawater 2.90 µg/L of beryllium. Additionally, the sorbent exhibited good reusability, as the modified MCM-41 was able to quantitatively extract Be^2+^ ions for more than six cycles.

Hasegawa utilized silica gel bonded with benzenesulfonic acid (SCX) in a column-based SPE setup for the determination of trace elements in pure molybdenum [106] and pure tungsten [107] samples using ICP-MS. The solid samples were decomposed with nitric acid for molybdenum and a mixture of HF, HNO_3_, and H_2_SO_4_ for tungsten, followed by the addition of hydrogen peroxide to promote anionization. The cationic trace impurities were retained by the ion-exchange sorbent and then eluted with diluted nitric acid. Both methods were optimized to detect several trace elements, including beryllium. The LOD varied between the two analyses: 0.11 ng/g for tungsten [107] and 0.028 ng/g for molybdenum [106].

#### 3.3.2. Carbon-Based Sorbents

Carbon-based materials are increasingly popular as sorbents in sample preparation due to their high surface area-to-volume ratios, high affinity for organic and inorganic molecules, metal ions, and gases, good physical and chemical stability, and low cost [108]. Among them, activated carbon, fullerenes, carbon nanotubes, graphene, and graphene oxide have been predominantly used in analytical applications.

Activated carbon (AC), composed of graphite crystallites, features a heterogeneous and irregular surface. It is produced through the pyrolysis or chemical treatment of materials such as nutshells, wood, and coal. The synthesis method and precursor material significantly influence its surface structure [109].

To separate and preconcentrate beryllium, AC suspensions have been applied to different matrices, including solid samples like coal, fly ash, and soil [110], as well as various water samples such as river, thermal, and freshwater [111], along with natural and flavored mineral water samples [12], prior to quantification by FAAS. Among the complexing agents tested—acetylacetone with morin, oxine, 1-(2-pyridylazo)-2-naphthol (PAN), and 4-(2-pyridylazo)resorcinol (PAR), the highest extraction recoveries (>85%) were achieved with a mixture of acetylacetone and morin [110]. This complexing agent was also used in studies by Avci [111] and Kilinç [12]. Procedure optimization led to excellent LOD of 0.010 µg/L [12] and 0.012 µg/L [111], resulting in a final preconcentration factor of 500. This improvement in detection allowed for the reliable quantification of ultra-trace beryllium concentrations in natural mineral water (0.05–0.94 µg/L), flavored mineral water (0.05–0.20 µg/L) [12], river water (0.07–0.183 µg/L), and freshwater (0.050–0.064 µg/L) [111]. Higher beryllium concentrations (0.815–1.687 µg/L) were detected in thermal water samples [111]. The method’s LOD (0.12 µg/L), though somewhat higher, was still sufficient for the quantification of beryllium in solid samples, where beryllium concentrations ranged from 284 to 375 ng/g in soil, 2353 to 3947 ng/g in fly ash, and 417 to 845 ng/g in coal [110].

Graphene, a single layer of carbon atoms arranged in a two-dimensional honeycomb lattice, serves as the fundamental building block of other carbon-based materials like graphite, carbon nanotubes, and fullerenes. Its exceptional electrical and thermal conductivity, mechanical strength, flexibility, and transparency make it valuable in various applications, including electronics, sensors, and batteries. In analytical chemistry, graphene has recently been adopted more widely as a sorbent due to its large surface area, strong adsorption ability, and chemical stability. However, challenges such as aggregation and poor dispersibility limit its direct use in analytical extraction. To address these issues, graphene derivatives such as graphene oxide (GO), reduced graphene oxide (rGO), and functionalized reduced graphene oxide (frGO) have been developed [112]. Functionalized graphene enhances selectivity for specific applications. For instance, tannic acid-functionalized graphene (TAFG) was used in a vortex-assisted DSPE (VA-DSPE) for beryllium separation and preconcentration from water and street dust samples [19]. Using ETAAS detection, an LOD of 0.84 ng/L was achieved. While beryllium levels in water samples were below the LOD, street dust contained 37.6 ± 0.3 ng/g of beryllium.

A dispersive micro-solid-phase extraction (D-µSPE) using graphene oxide (GO) has been developed to extract trace amounts of beryllium, generated by beryllium processing plants, from wastewater [113]. In this approach, GO serves as a micro-sorbent, while 1-octanol acts as an extracting agent. GO nanosheets are dispersed in alkaline wastewater, where they efficiently capture Be^2+^ ions. To facilitate the separation and preconcentration of the beryllium-bound GO, sodium chloride is added, which neutralizes GO’s surface charge, reducing its hydrophilicity and inducing aggregation. This allows GO to be easily extracted into a small volume of 1-octanol through hydrophobic interactions. Beryllium is then recovered from 1-octanol using ultrasonic extraction with diluted nitric acid and quantified via ETAAS. Under optimized conditions, this method achieves a preconcentration factor of 100 and an LOD of 2 ng/L.

#### 3.3.3. Magnetic Sorbents

Magnetic solid-phase extraction (MSPE) is a variant of SPE conducted in dispersion mode using magnetic sorbents. This technique has gained considerable attention in sample pretreatment due to its unique magnetic features, ease of implementation, and cost-effectiveness. In MSPE, an external magnet is employed to isolate the magnetic sorbents loaded with target analytes, offering a simpler and more efficient alternative to conventional methods such as centrifugation or filtration.

A diverse range of magnetic sorbents has been developed for extracting various analytes from different matrices. Notable examples include Fe_3_O_4_-based materials such as metal–organic frameworks (Fe_3_O_4_@MOFs), covalent organic frameworks (Fe_3_O_4_@COFs), magnetic molecularly imprinted polymers (MMIPs), magnetic ion-imprinted polymers (MIIPs), and magnetic carbon materials (MCMs) [108]. Some of these materials have also been explored for the separation and preconcentration of beryllium.

One such application involved the synthesis of a magnetic metal–organic framework (MOF) using magnetite (Fe_3_O_4_) nanoparticles modified with 2-amino-5,8-dihydroxy-1,4-naphthoquinone (ADHNQ). These modified nanoparticles were then reacted with terephthalic acid and iron chloride to form a MIL-53(Fe)-type MOF (Fe_3_O_4_@SiO_2_@ADHNQ/MIL-53(Fe)). This material was successfully employed for the extraction and preconcentration of beryllium ions, followed by quantification using FAAS [10]. Optimization of the procedure yielded an LOD of 0.07 µg/L and an LOQ of 0.20 µg/L. The method’s analytical applicability was assessed through the analysis of seawater, well water, river water, and Cu–Be alloy samples. While beryllium concentrations in well water and river water were below the LOD, Caspian seawater contained 1.7 ± 0.05 µg/L of beryllium, and Cu–Be alloy samples had 0.64 ± 0.04%.

The use of magnetic ion-imprinted polymers (MIIPs) for the simultaneous separation and preconcentration of aluminum and beryllium from water samples was reported relatively recently [114]. Two new MIIPs were synthesized using chrome azurol S (CAS) as the ligand, (3-aminopropyl)triethoxysilane (APTES) as the functional monomer, tetraethyl orthosilicate (TEOS) as the cross-linker, with aluminum and beryllium ions serving as templates. These MIIPs functioned as magnetic sorbents. The optimized MSPE procedure was coupled with a spectrophotometric method using the mean centering of ratio (MCR) approach for quantification. For beryllium, an LOD of 0.90 µg/L was achieved. The method’s performance was evaluated by applying it to tap and river water, though beryllium concentrations in these real-world samples were below the LOD. To assess accuracy, the samples were spiked with different beryllium concentrations, demonstrating the method’s suitability for water analysis across varying compositions and sources.

#### 3.3.4. Other Sorbents

This section will provide a discussion on traditional column-based SPE setups utilizing various sorbent materials, including Oasis HLB, strong-base anion-exchange resin, chelating ion exchangers, and human hair. Additionally, we will cover a specialized technique, a syringe membrane SPE (SMSPE), which employs a TiO_2_@SiO_2_ flexible nanofiber membrane.

A laboratory-prepared Oasis HLB cartridge was used to separate and preconcentrate beryllium from liver and muscle samples (bovine, chicken, and pork) after their decomposition with a mixture of HNO_3_ and H_2_O_2_ [115]. Oasis HLB is a patented polymeric reversed-phase sorbent with both hydrophilic and lipophilic retention properties. ‘HLB’ stands for hydrophilic–lipophilic balance, referring to the sorbent’s ability to (1) remain water-wettable and (2) retain a broad range of polar and non-polar compounds. In this method, beryllium was complexed with acetylacetone (Be(acac)_2_), retained on the sorbent, and then eluted with methanol. A portion of the eluted solution was introduced into a graphite furnace for atomization using an optimized temperature program. This method achieved an LOD of 0.18 ng/g and an LOQ of 0.60 ng/g, enabling the detection of beryllium concentrations ranging from 2.3 to 4.7 ng/g in the analyzed samples.

For the selective binding of beryllium ions in mineral water, a specific type of ion-exchange resin with chelation properties (Ostsorb Salicyl) was used [116]. As part of the pretreatment process, EDTA was added to mask the interference of alkaline Earth metals and iron. The optimized extraction procedure was then combined with two detection techniques: ETAAS and ICP-OES, achieving LODs of 0.01 µg/L and 0.25 µg/L, respectively. These low LODs enabled the quantification of beryllium in natural mineral water samples from the Czech Republic, in a concentration range of 0.07 to 94.5 µg/L.

The preconcentration of beryllium ions using a strong-base anion-exchange resin is another example of an SPE technique based on a column setup [117]. After retaining the beryllium on the resin, it was eluted with 1.5 M HCl and measured using FAAS. After optimization, the method achieved an LOD of 0.045 µg/L and was successfully applied to spring, river, and wastewater samples. Interestingly, beryllium concentrations in spring and wastewater samples were similar, 0.17 and 0.15 µg/L, respectively, while river water contained higher levels (~0.35 µg/L). However, the study did not specify the sampling locations, leaving the source of these variations unclear.

An unconventional sorbent, human hair, was used to adsorb beryllium prior to its spectrophotometric quantification [62]. A custom-made column was filled with 1.0 g of human hair, which had been washed with synthetic detergent, then rinsed with deionized water and 0.5 M HNO_3_. After optimization, the method enabled the detection of ultra-trace beryllium levels. In canal water samples, the mean concentration was 0.22 µg/L, while in tap water samples, it was 0.12 µg/L. The detection of these concentrations was made possible due to an enrichment factor of 50, leading to an LOD of 0.028 µg/L.

Recently, an efficient method for quantifying beryllium in Chinese yellow rice wines was reported by Wang [20]. In this approach, wine samples were first diluted with ultra-pure water, then preconcentrated using syringe membrane SPE (SMSPE), followed by detection via ETAAS. The sorbent used in the SMSPE process was a TiO_2_@SiO_2_ flexible nanofiber membrane (TSFNFM), which effectively enriched beryllium while simultaneously removing interfering matrix components. With an LOD of 0.52 ng/L, this method was highly sensitive for detecting beryllium concentrations, which ranged from 0.09 to 0.41 µg/L in wine samples. Notably, the reusability of the sorbent was investigated, demonstrating that its extraction efficiency remained above 90% for at least 12 cycles, confirming TSFNFM’s potential for repeated use without significant loss of performance (sorption ability).

## 4. Conclusions

The quantification of beryllium presents significant challenges due to the complexities associated with analyzing this element at trace levels, potential interferences from other components in the sample matrix, its tendency to form stable complexes, adsorption losses, and the limited sensitivity of certain analytical methods. This study has thoroughly examined various spectrometric techniques available for beryllium analysis, including flame atomic absorption spectrometry (FAAS), electrothermal atomic absorption spectrometry (ETAAS), inductively coupled plasma optical emission spectrometry (ICP-OES), inductively coupled plasma mass spectrometry (ICP-MS), X-ray fluorescence (XRF), laser-induced breakdown spectroscopy (LIBS), and UV-Vis spectrophotometry (UV-Vis). While these methods vary in sensitivity and applicability, they share common obstacles that must be addressed to ensure accurate quantification of beryllium in diverse matrices. Research on trace toxic element analysis often involves monitoring several toxic elements concurrently. This approach usually enables better characterization and correlation of data, making techniques like ICP-MS and ICP-OES particularly attractive due to their versatility and sensitivity. One of the primary challenges in quantifying beryllium using ICP-MS is managing matrix effects caused by variations in sample composition, which can impact ionization efficiency and signal stability. While beryllium’s low mass minimizes isobaric interferences, polyatomic interferences such as ^1^H^8^O^+^ and matrix effects must be carefully addressed during analysis. The low atomic number of beryllium (4) makes it particularly susceptible to space-charge effects, where the presence of heavier elements in a sample can reduce the transmission efficiency of lighter elements like beryllium, leading to a significant reduction in sensitivity and accuracy. This phenomenon can complicate the reliable detection of beryllium, especially in complex natural samples where matrix interference is prevalent. For instance, while ICP-MS can achieve detection limits as low as 0.3 ng/L for beryllium, the presence of high salt concentrations or other competing species can drastically impair performance, necessitating effective separation and preconcentration techniques.

While ETAAS offers higher sensitivity than FAAS, with limits of detection (LOD) reaching approximately 0.02 µg/L when combined with cloud-point extraction (CPE), it still faces limitations related to reproducibility and memory effects. Modifications such as the use of pyrolytically coated graphite tubes can improve performance, but these solutions do not entirely eliminate the issues associated with method reliability.

Direct measurement of beryllium by X-ray fluorescence (XRF) is problematic due to beryllium’s low atomic number, which results in reduced sensitivity compared to heavier elements. Indirect methods have been proposed to address this issue, but they often require specialized instrumentation and careful sample preparation to achieve acceptable accuracy. This challenge is further compounded by the fact that XRF generally offers lower sensitivity than other techniques, making it unsuitable for ultra-trace analysis.

Even in techniques like UV-Vis spectrophotometry, where the formation of colored complexes is essential for detection, the requirement for specific reagents and optimal conditions can complicate the process. For example, using chrome azurol S (CAS) as a reagent has been effective for high beryllium concentrations, but its applicability is limited to simpler matrices, and sample preparation can introduce variability in the results.

Liquid-liquid extraction (LLE) and solid-phase extraction (SPE) have proven effective in addressing these challenges. However, the classic LLE techniques that previously relied on large volumes of hazardous solvents have been phased out in favor of greener alternatives, such as dispersive liquid-liquid microextraction (DLLME) and single-drop microextraction (SDME). These modern techniques combined with spectrometric detection achieve impressive enhancement factors, with some methods reporting LODs as low as 8.0 ng/L for beryllium, but they still necessitate careful optimization and validation in real-world scenarios to ensure reliability.

Additionally, the quantification of beryllium in environmental samples, such as seawater, wastewater, and various solid matrices, is particularly challenging due to the trace concentrations typically encountered. For instance, beryllium concentrations in seawater have been reported as low as 0.13 µg/L, while effluent samples can vary significantly, ranging from 9 to 92 µg/L. The inherent variability in sample composition demands robust analytical protocols and an understanding of the potential interferences that may arise during analysis.

As anthropogenic activities continue to impact the natural environment, the demand for reliable and sensitive methods to quantify beryllium is more urgent than ever. The synthesis and application of novel sorbents, such as those based on graphene oxide or magnetic materials, represent promising advancements in addressing the challenges of beryllium quantification. Nevertheless, the ongoing development of analytical methods must prioritize overcoming the limitations associated with matrix effects, detection sensitivity, and method reproducibility.

In summary, while significant progress has been made in the quantification of beryllium, the challenges presented by its low concentration, susceptibility to interference, and the complexities of sample matrices necessitate continued research and innovation in analytical methodologies. By enhancing the accuracy and reliability of beryllium measurement techniques, we can better assess the environmental and health risks associated with this toxic element, ultimately contributing to more effective regulatory measures and public health initiatives.

## Figures and Tables

**Table 1:** Comparison of spectroanalytical techniques for beryllium quantification based on sensitivity, specificity, and susceptibility to interferences. The techniques are roughly ordered by their typical detection ranges, though actual performance may vary due to element-specific factors, matrix effects, and analytical conditions.

Technique	Typical Detection Range	Sensitivity	Specificity	Matrix Interference Susceptibility	Advantages	Disadvantages
ICP-MS	ppt to % levels	very high	very high	low	extreme sensitivity (ppt range), isobaric interferences for Be are generally low, multi-element detection	expensive instrumentation, high maintenance, Be’s high ionization potential (9.3 eV) → ~70–75% ionization in Ar plasma, space-charge effects within the ion beam
ETAAS	ppb levels	high	high	moderate	high sensitivity (ppb range) for Be, small sample volume required	longer analysis time than FAAS, requires precise temperature control to avoid Be loss, more complex sample preparation
FS	ppb to ppm levels	moderate	low	high	high sensitivity when Be forms fluorescent complexes with organic reagents	highly susceptible to matrix effects, requires derivatization
ICP-OES	ppb to % levels	high	high	moderate to low	multi-element analysis, high throughput	relatively costly (although less than ICP-MS), susceptible to matrix effects
FAAS	ppm levels	moderate	moderate	moderate	simple, cost-effective	limited sensitivity for Be (ppm range), matrix effects
UV-Vis	ppm levels	low to moderate	low	high	inexpensive, simple operation	limited sensitivity for Be, requires complexation with organic ligands for Be detection, strong matrix effects
LIBS	ppm to % levels	moderate	moderate	moderate	fast, minimal sample preparation	limited sensitivity for Be due to low emission intensity, susceptible to matrix effects
XRF	ppm to % levels	moderate	moderate to high	low	minimal sample preparation, non-destructive	poor sensitivity for Be due to its low atomic number, less effective for light elements like Be

ICP-MS: inductively coupled plasma mass spectrometry; ETAAS: electrothermal atomic absorption spectrometry; FS: fluorescence spectroscopy; ICP-OES: inductively coupled plasma optical emission spectrometry; FAAS: flame atomic absorption spectrometry; UV-Vis: UV-Vis spectrophotometry; LIBS: laser-induced breakdown spectroscopy; XRF: X-ray fluorescence.

## Data Availability

The original contributions presented in this study are included in the article. Further inquiries can be directed to the corresponding author.
